# Lysis-deficient phages as novel therapeutic agents for controlling bacterial infection

**DOI:** 10.1186/1471-2180-11-195

**Published:** 2011-08-31

**Authors:** Vivek Daniel Paul, Sudarson Sundarrajan, Sanjeev Saravanan Rajagopalan, Sukumar Hariharan, Nanjundappa Kempashanaiah, Sriram Padmanabhan, Bharathi Sriram, Janakiraman Ramachandran

**Affiliations:** 1 Gangagen Biotechnologies Pvt Ltd, No. 12, 5th Cross, Raghavendra Layout, Tumkur Road, Yeshwantpur, Bangalore-560 022, India; 2 Department of Molecular Genetics, University of Toronto, 1 King's College Circle, Toronto, ON M5S 1A8, Canada; 3 Department of Animal Husbandry, Veterinary Dispensary, Yediyur, Kunigal Taluk, Tumkur- 572142, India; 4 Lupin Limited, Biotechnology R & D, Gat #1156, Ghotawade Village, Mulshi Taluka, Pune-411042, India

## Abstract

**Background:**

Interest in phage therapy has grown over the past decade due to the rapid emergence of antibiotic resistance in bacterial pathogens. However, the use of bacteriophages for therapeutic purposes has raised concerns over the potential for immune response, rapid toxin release by the lytic action of phages, and difficulty in dose determination in clinical situations. A phage that kills the target cell but is incapable of host cell lysis would alleviate these concerns without compromising efficacy.

**Results:**

We developed a recombinant lysis-deficient *Staphylococcus aureus *phage P954, in which the endolysin gene was rendered nonfunctional by insertional inactivation. P954, a temperate phage, was lysogenized in *S. aureus *strain RN4220. The native endolysin gene on the prophage was replaced with an endolysin gene disrupted by the chloramphenicol acetyl transferase (*cat*) gene through homologous recombination using a plasmid construct. Lysogens carrying the recombinant phage were detected by growth in presence of chloramphenicol. Induction of the recombinant prophage did not result in host cell lysis, and the phage progeny were released by cell lysis with glass beads. The recombinant phage retained the endolysin-deficient genotype and formed plaques only when endolysin was supplemented. The host range of the recombinant phage was the same as that of the parent phage. To test the *in vivo *efficacy of the recombinant endolysin-deficient phage, immunocompromised mice were challenged with pathogenic *S. aureus *at a dose that results in 80% mortality (LD_80_). Treatment with the endolysin-deficient phage rescued mice from the fatal *S. aureus *infection.

**Conclusions:**

A recombinant endolysin-deficient staphylococcal phage has been developed that is lethal to methicillin-resistant *S. aureus *without causing bacterial cell lysis. The phage was able to multiply in lytic mode utilizing a heterologous endolysin expressed from a plasmid in the propagation host. The recombinant phage effectively rescued mice from fatal *S. aureus *infection. To our knowledge this is the first report of a lysis-deficient staphylococcal phage.

## Background

Bacteriophages are attractive as therapeutic agents because they are safe for humans and highly specific and lethal to the bacteria they target. Further, phages can be developed rapidly to combat the emergence of antibiotic-resistant pathogenic bacteria [1,2]. Phage therapy is currently practiced routinely and successfully in countries such as Poland and Russia [3]. The recent approval of commercial phage preparations by the United States Food and Drug Administration to prevent bacterial contamination of meat and poultry [4] may pave the way for the global use of phage therapy to control bacteria in human infections.

The development of phages for therapy has been hampered by concerns over the potential for immune response, rapid toxin release by the lytic action of phages, and difficulty of dose determination in clinical situations [5]. Phages multiply logarithmically in infected bacterial cells, and the release of progeny phage occurs by lysis of the infected cell at the end of the infection cycle, which involves the holin-endolysin system [6,7]. Holins create a lesion in the cytoplasmic membrane through which endolysins gain access to the murein layer [7]. Endolysins are peptidoglycan hydrolases that degrade the bacterial cell wall, leading to cell lysis and release of progeny phages [8]. An undesirable side effect of this phenomenon from a therapeutic perspective is the development of immunogenic reactions due to large uncontrolled amounts of phages in circulation [9]. Such concerns must be addressed before phage therapy can be widely accepted [5,10].

This work features engineered bacteriophages that are incapable of lysing bacterial cells because they lack endolysin enzymatic activity. We previously produced, as a model, a recombinant lysis-deficient version of T4 bacteriophage that infects *Escherichia coli *[11,12]. Phages have also been engineered to be non- replicating or to possess additional desirable properties [13-15]. In an experimental *E. coli *infection model, the improved survival rate of rats treated with lysis-deficient T4*LyD *phage was attributed to lower endotoxin release [16].

We wished to generate an endolysin-deficient phage against a gram-positive bacterium, and chose *S. aureus *because of its clinical relevance. *S. aureus *is a major pathogen responsible for a variety of diseases ranging from minor skin infections to life-threatening conditions such as sepsis. This pathogen is often resistant to all β-lactam antibiotics; vancomycin-resistant strains may become untreatable [17-19]. This organism is the most common cause of nosocomial infections, and nasal carriage is implicated as a risk factor [20]. In the United States alone, invasive methicillin-resistant *S. aureus *(MRSA) infections occur in approximately 94,000 people each year, causing nearly 19,000 deaths [21]. Understandably, the progressive multidrug resistance of bacteria has motivated the re-evaluation of phages as therapy for diverse bacterial infections [22].

We report here that the recombinant endolysin-deficient *S*. *aureus *phage P954 kills cells without causing cell lysis and forms plaques on a host that expresses a plasmid-encoded heterologous endolysin, enabling its large-scale production. The recombinant phage P954 was evaluated for *in vivo *efficacy in an experimental mouse model and found to protect mice from fatal *S. aureus *infection.

## Methods

### Bacterial strains, plasmids, and growth conditions

*E. coli *strain DH5α [Φ80d*lacZΔM15 *Δ (*lacZYA-argF*) *recA1 endA1 hsdR17 supE44 thi-1 gyrA96relA1deoR*] was used as host for plasmid constructions and plasmid propagation. A restriction-deficient prophage-free *S. aureus *strain RN4220 [23] was used for recombination, lysogenization, and phage enrichment. Clinical isolates of *S. aureus *were used to test phage sensitivity. A MRSA clinical isolate (B911) was used in animal experiments to determine the *in vivo *efficacy of the endolysin-deficient phage P954.

The plasmid pET21a (Novagen, USA) was used for cloning and construction of endolysin disruption cassette. The plasmid pSK236, an *E. coli - S. aureus *shuttle vector containing pUC19 cloned into the HindIII site of *S. aureus *plasmid pC194 [24], was used as a source for the *cat *gene. A shuttle vector containing the temperature-sensitive replication origin of *S. aureus*, pCL52.2, was used as source for the replication origin [25]. The constitutive *Bacillus subtilis vegII *promoter was derived from pRB474 [26]. All bacterial strains were cultured in liquid Luria Bertani (LB) medium at 37°C on a rotary shaker (200 rpm) unless otherwise stated. Ampicillin, chloramphenicol, and tetracycline were used as needed. All chemicals were obtained from Sigma-Aldrich, St. Louis, MO, USA unless otherwise mentioned.

### Propagation, concentration, and enumeration of bacteriophages

Bacteriophage P954 is a temperate phage that was isolated from the Ganges River (India) and amplified in *S. aureus *strain RN4220. Briefly, *S. aureus *RN4220 was grown at 37°C in LB medium to an absorbance of approximately 0.8 at 600 nm, infected with phage P954 at a multiplicity of infection (MOI) of 0.01, and cultured at 37°C until the culture lysed completely. After centrifugation at 4100 × g for 10 min to remove cell debris, the bacteriophages were concentrated by centrifugation at 27,760 × g for 90 min. The bacteriophage titer was determined by enumerating plaque-forming units (PFUs) in serial 10-fold dilutions in LB medium and confirmed by the agar overlay method [27,28].

### Preparation of phage P954 DNA and genome sequencing

Phage P954 DNA was prepared from a stock solution (1 × 10^12 ^PFU/ml). The concentrated phage preparation (1 ml) was incubated at 37°C for 1 hr with DNase I (1 μg/ml) and RNase A (100 μg/ml). The mixture was adjusted to contain 1% sodium dodecyl sulfate, 50 mM EDTA (pH 8.0), and 0.5 μg proteinase K and incubated at 65°C for 60 min. The mixture was then subjected to phenol-chloroform-isoamyl alcohol (25:24:1) extraction, and the DNA was precipitated [29]. Purified phage DNA was used for genome sequencing [GenBank: GQ398772].

### Construction of plasmids for phage P954 endolysin disruption

The phage P954 endolysin gene (753 bp) was amplified as two separate fragments by polymerase chain reaction (PCR). The first fragment (bp 1-376) was amplified with forward primer 5'-CGGAATTC*catatg*AAAACATACAGTGAAGCAAGAGCA-3', containing an NdeI restriction site, and reverse primer 5'-CCGCCGCT*gaattc*TAATAAAGTGAGTACAGCC-3', containing an EcoRI site. The fragment was cloned into a pET21a vector at the NdeI/EcoRI sites.

The second fragment (bp 377-753) was amplified with forward primer 5'-CCGCCGG*gaattc*AGTATAAAAGTGAGGGCTTA-3', containing an EcoRI site, and reverse primer 5'-CC*aagctt*TTAAAACACTTCTTTCACAATCAATCTCTC-3', containing a HindIII site. The second fragment was cloned in tandem with the first fragment, thus generating the full-length phage P954 lysin gene with an internal EcoRI site. The *cat *gene was isolated along with its constitutive promoter from the *S. aureus *- *E. coli *shuttle plasmid pSK236 by ClaI digestion. Cohesive ends were filled with the Klenow fragment of DNA polymerase I and ligated into the blunted EcoRI site of the full-length phage P954 endolysin gene, thereby disrupting it. The *S. aureus*-specific temperature-sensitive origin of replication from the shuttle vector pCL52.2 was introduced at the XhoI restriction site of this construct to generate pGMB390.

### Mitomycin C induction of phage P954 lysogens

The *S. aureus *RN4220 lysogen of phage P954 was inoculated in LB medium and incubated at 37°C with shaking at 200 rpm for 16 hr. The cells were then subcultured in LB medium at 2% inoculum and incubated at 37°C with shaking at 200 rpm until the culture attained an absorbance of 1.0 at 600 nm. Mitomycin C was then added to a final concentration of 1 μg/ml, and the culture was incubated at 37°C with shaking at 200 rpm for 4 hr for prophage induction.

### Recombination and screening for recombinants

*S. aureus *RN4220 cells were transformed with pGMB390 by electroporation according to the protocol described by Schenk and Laddaga [30] with a BioRad Gene Pulser, plated on LB agar containing chloramphenicol (10 μg/ml), and incubated at 37°C for 16 hr. Chloramphenicol-resistant colonies were selected and grown in LB at 37°C until the cultures reached an absorbance of 1.0 at 600 nm. Recombination was then initiated by infecting these cells with phage P954 (MOI = 3) for 30 min. Progeny phage were harvested from the lysate as described previously, lysogenized in *S. aureus *RN4220, and plated on LB agar containing chloramphenicol (10 μg/ml) (round I). Ninety-six chloramphenicol-resistant colonies were picked up, grown, and induced with Mitomycin C. Cultures that did not lyse after the 16-hr Mitomycin C induction were treated with 1% chloroform and lysed with glass beads; the released phages were again lysogenized in *S. aureus *RN4220 (round II). Chloramphenicol-resistant colonies of round II lysogens were similarly grown and subjected to Mitomycin C induction. The chloramphenicol-resistant lysogens that did not release phages upon Mitomycin C induction were selected for PCR analysis. Genomic DNA of the selected lysogens was purified, and PCR was performed with different sets of primers to confirm disruption of the phage P954 endolysin gene.

### Endolysin complementation for phage enrichment and enumeration

The endolysin gene from a Podoviridae phage in our collection, P926, was cloned under the constitutive *B. subtilis vegII *promoter in an *E. coli - S. aureus *shuttle vector constructed in our laboratory. This construct, designated pGMB540, was used for trans-complementation of the nonfunctional endolysin for propagation of the recombinant phage in lytic mode and for their enumeration. Plasmid pGMB540 was introduced into *S. aureus *strain RN4220 by electroporation according to the protocol described by Schenk and Laddaga [30]. Transformants were selected on LB medium containing tetracycline (5 μg/ml) and used as bacterial hosts for phage enrichment. Early log phase cells of *S. aureus *RN4220/pGMB540 grown at 37°C were infected with the recombinant endolysin-deficient phage P954 (MOI = 0.1) and incubated for an additional 3 to 4 hr until the culture lysed. The phage-containing lysate was passed through a 0.2-μm filter, and the phages were enumerated on a lawn of *S. aureus *RN4220/pGMB540 cells.

The endolysin-deficient phage P954 was also enriched by induction. Briefly, the lysogen was grown at 37°C until absorbance at 600 nm reached 1.0 and then induced with 1 μg/ml Mitomycin C at 37°C for 4 hr. The cells were pelleted and lysed by vortexing with glass beads. Cell debris was removed by centrifugation at 5000 × g for 10 min, and the phage-containing supernatant was passed through a 0.2-μm filter.

### Comparison of *in vitro *bactericidal activity of parent and lysis-deficient phage P954

The parent and recombinant phages were compared for host range and bactericidal activity. Ten MOI equivalent of phage was added to 2 × 10^8 ^colony-forming units per ml (CFU/ml) and incubated at 37°C for 90 min. Serial 10-fold dilutions of the mixture were plated on LB agar, and residual viable cells (CFUs) were enumerated.

### *In vivo *efficacy of endolysin-deficient phage P954 in neutropenic mice

Animal experiments were performed at St. John's Medical College and Hospital, Bangalore, India. The experiments were approved by the Institutional Animal Ethics Committee and the Committee for the Purpose of Control and Supervision of Experiments on Animals (registration No. 90/1999/CPCSEA dated 28/4/1999).

Healthy male Swiss albino mice (6-8 weeks old, neutropenic) were used to evaluate *in vivo *efficacy. Neutropenia was induced by intraperitoneal (IP) administration of cyclophosphamide (100 mg/kg). In a preliminary study, the lethality of a clinical MRSA isolate (B911) was determined in the mice (1 × 10^7 ^-1 × 10^8 ^CFU). We found that 5 × 10^7 ^CFU resulted in 80% mortality (LD_80_), and it was therefore chosen as the challenge dose to evaluate phage efficacy (data not shown).

In the efficacy experiment, mice were assigned to six treatment groups (n = 8, each group). Four days after cyclophosphamide treatment, the mice in groups 1-3 were challenged with B911 (200 μl, 5 × 10^7 ^CFU). Groups 1 and 4 were then treated with 25 mM Tris-HCl, pH 7.5 (negative control); groups 2 and 5 were treated with two doses of endolysin-deficient phage P954 prepared in 25 mM Tris-HCl, pH 7.5 at 200 MOI equivalent (MOI relative to CFU at LD_80_); and groups 3 and 6 were treated with two doses of chloramphenicol (50 mg/kg). The first treatment dose was administered immediately after challenge; the second dose was administered 2 hr later. Mice were observed over 10 days for occurrence of mortality. Survival analysis is plotted as Kaplan-Meier survival curves using MedCalc statistical software version 11.6.0.0 (Mariakerke, Belgium).

## Results

### Genome of phage P954

The 40761-bp phage P954 genome (Genome map provided as Additional file 1 Figure S1) is composed of linear double-stranded DNA with a G+C content of 33.99% [GenBank: GQ398772]. BlastN [31] searches with the phage P954 nucleotide sequence showed it to be similar to other sequenced staphylococcal phages in the NCBI database. The P954 genome matches that of *S. aureus *phage phiNM3 (accession no. DQ530361) with pair-wise identity of 66%. At least 69 open reading frames (ORFs) were predicted with the GeneMark program [32]. Bioinformatics analysis revealed that 46 of the 69 ORFs are hypothetical/conserved hypothetical proteins; the other 23 ORFs show a high degree of homology to proteins from other staphylococcal phages in the database. The lysis cassette of this phage was found to be similar to lysis systems of other staphylococcal phages. The closest match to the phage P954 holin gene was staphylococcal prophage phiPV8, with 97% identity. The endolysin gene of phage P954 is 100% identical to the amidase gene from staphylococcal phage phi13; the phage P954 integrase gene is 100% identical to ORF 007 of staphylococcal phage 85; and the phage P954 repressor gene is 100% identical to the putative phage repressor of *S. aureus *subsp JH9. Our analysis did not reveal the presence of any toxin encoding genes in the phage P954 genome.

### Screening of recombinants

**Figure 1:**
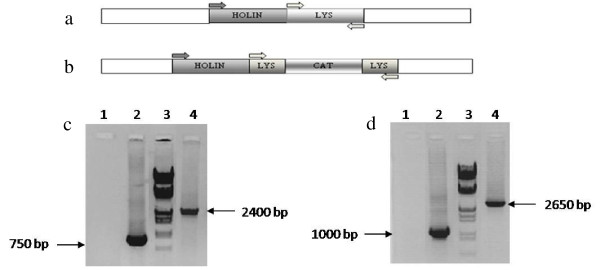
**Schematic and PCR analysis of parent and recombinant endolysin-deficient phage P954**. Alignment of primers to the (**a) **parent phage DNA template and **(b) **recombinant phage DNA template, in which the *cat *gene had been inserted into the endolysin gene (LYS). **(c) **Endolysin forward and reverse primers yield a 750-bp PCR product of the parent phage P954 and 2400-bp product of the recombinant phage P954. **(d) **The holin forward primer and endolysin reverse primer yield a 1000-bp PCR product with parent phage P954 and 2650-bp product of the recombinant phage P954. Both PCR panels include lane 1: PCR buffer (negative control); lane 2: parent phage P954 lysogen B7, lane 3: molecular weight marker (λ/HindIII-EcoRI); lane 4: recombinant phage P954 lysogen H10.

The native phage endolysin gene was inactivated, and the recombinant phage engendered by homologous recombination between phage P954 and plasmid pGMB390 in *S. aureus *RN4220. Screening for *S. aureus *RN4220 lysogens harboring recombinant phage P954, in which endolysin was inactivated by insertion of the *cat *gene, was carried out using chloramphenicol resistance as a marker. Ninety-six colonies were obtained of which two lysogens did not show lysis with Mitomycin C induction for up to 16 hours. Phages mechanically released from these colonies upon relysogenization yielded chloramphenicol resistant lysogens that did not lyse upon Mitomycin C induction. PCR analyses using two primer sets confirmed disruption of the endolysin gene in all the recombinant lysogens screened. Representative PCR profile of recombinant and parent phage lysogens is shown (Figure 1).

### Mitomycin C induction of parent and endolysin-deficient phage P954

**Figure 2:**
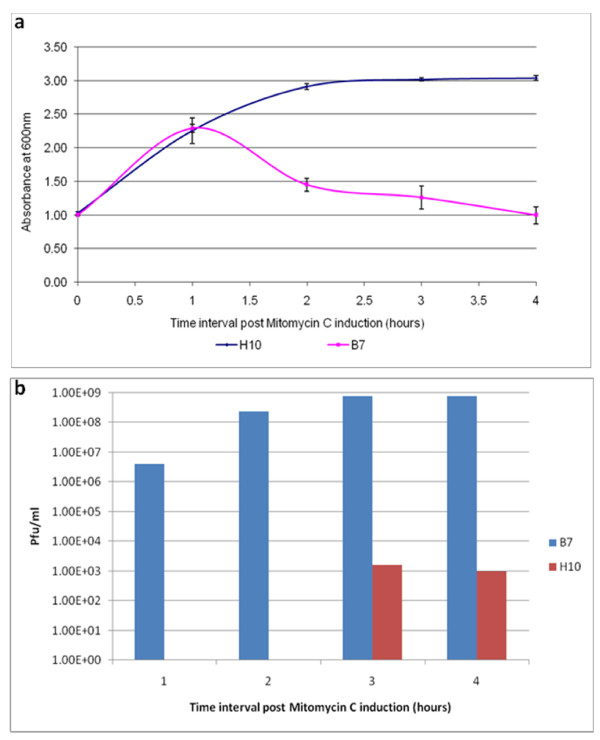
**Mitomycin C induction of parent and endolysin-deficient phage P954 lysogens**. **(a) **Growth profiles of the parent (B7) and endolysin-deficient (H10) phage P954 lysogens after Mitomycin C induction showing absorbance of cultures at 600 nm. The graph is representative of two experiments. The error bars represent mean plus standard deviation (n = 3) **(b) **Phage release into the culture medium from parent (B7) and endolysin-deficient (H10) phage P954 lysogens after Mitomycin C induction. The graph is representative of 2 experiments.

We examined the prophage induction pattern and phage progeny release from parent and endolysin-deficient phage P954 lysogens. Absorbance and extracellular phage titers were monitored every hour until the end of induction. Induction of the parent phage P954 lysogen (B7) resulted in cell lysis and gave a phage titer of 1 × 10^9 ^PFU/ml. In contrast, the endolysin-deficient phage P954 lysogen did not lyse and gave a phage titer of about 10^3 ^PFU/ml (Figure 2).

### Endolysin complementation for phage enrichment and enumeration

**Figure 3:**
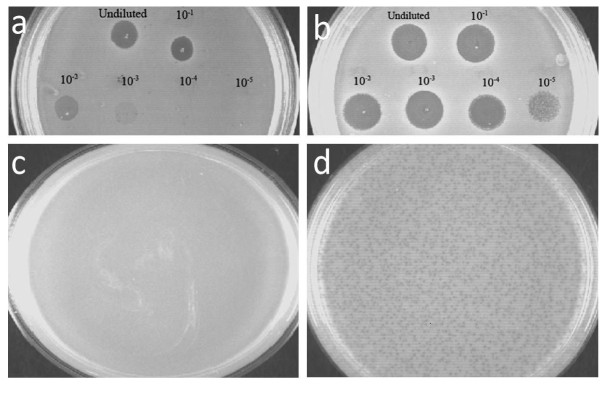
**Complementation with heterologous endolysin gene for enrichment of endolysin-deficient phage P954**. Ten-fold serial dilutions of endolysin-deficient phage P954 (5 × 10^10 ^PFU/ml) spotted on **(a) ***S. aureus *RN4220 lawn and **(b) **complementing host pGMB540/*S. aureus *RN4220, which expresses a heterologous endolysin. Plaque assay of enriched endolysin-deficient phage P954 on **(c) **non-complementing host *S. aureus *RN4220 and **(d) **complementing host pGMB540/*S. aureus *RN4220.

Endolysin-deficient phage P954 could be enriched to titers of up to 5 × 10^10 ^PFU/ml in *S. aureus *RN4220 that constitutively expressed phage P926 endolysin. This strain was used also to determine titers of the endolysin-deficient phage preparations. When preparations of the endolysin-deficient phage were spotted on a non-complementing host, a zone of lysis characteristic of "lysis from without" was observed at lower dilutions, and no plaques were discernible (Figure 3a). The recombinant phage formed plaques only on the endolysin-complementing host (Figure 3b, c, d).

### Comparison of *in vitro *bactericidal activity of parent and lysis-deficient phage P954

**Figure 4:**
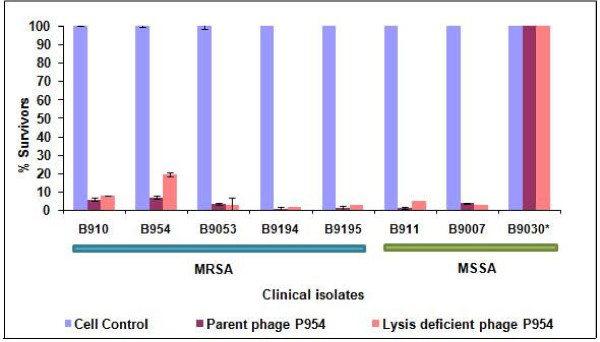
**Bactericidal activity of parent and lysis-deficient phage P954**. Bactericidal activity of parent and lysis-deficient phage P954 (10 MOI equivalent) on eight clinical isolates of MRSA (B910, B954, B9053, B9194, B9195) and MSSA (B911, B9007, B9030). Phage resistant isolate indicated with asterix (*). The error bars represent standard deviation (n = 3, single experiment).

Parent and recombinant endolysin-deficient phage P954 demonstrated comparable bactericidal activity when tested on a panel of seven phage-sensitive and one phage-resistant (B9030) clinical isolates, comprising both methicillin-sensitive *S. aureus *(MSSA) and MRSA strains, from our collection. Cell viability was reduced by ≥ 90% with both phages (Figure 4). Similarly, the host range of each phage was the same on a panel of 20 phage-sensitive and phage-resistant clinical isolates (data provided as Additional file 2 Table S1).

### *In vivo *efficacy of endolysin-deficient phage P954

**Figure 5:**
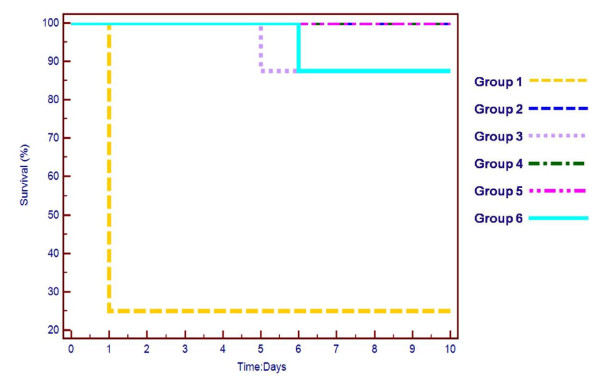
***In vivo *efficacy of endolysin-deficient phage P954**. Survival of mice challenged with clinical MRSA isolate (B911). Groups 1-3 were challenged with MRSA (5 × 10^7 ^cells per mouse). Groups 4-6 were not challenged with MRSA and served as controls. The following treatments were administered: groups 1 and 4 (25 mM Tris-HCl, pH 7.5); groups 2 and 5 (two doses of endolysin-deficient phage P954, 200 MOI); groups 3 and 6 (two doses of chloramphenicol, 50 mg/kg).

An IP injection of the MRSA isolate B911 (5 × 10^7 ^cells/mouse) resulted in the onset of disease in 80% of mice (group 1), indicated by dullness, ruffled fur, and death within 48 hr (Figure 5). However, IP administration of endolysin-deficient phage P954 as two doses (immediately and after 2 hr) post B911 challenge fully protected the mice against lethality (group 2). Similarly, chloramphenicol (dose regimen similar to phage) protected mice against lethality (group 3); however, one animal died in each of the chloramphenicol treatment groups of unknown causes (groups 3 and 6). Endolysin-deficient phage alone was not toxic or lethal to neutropenic mice, demonstrating its safety (group 5). Endolysin-deficient phage demonstrated significant efficacy against MRSA B911in the tested animal model (P value = 0.0001).

## Discussion

Bacteriophage endolysins are peptidoglycan hydrolases that function at the end of the phage multiplication cycle, lysing the bacterial cell and releasing new phages to infect other bacteria. Many efforts to develop therapeutic phages have focused on the lytic endpoint of phage infection to destroy the bacterium. However, cell lysis by phage may present the problem of endotoxin release and serious consequences as known in the case of antibiotics [33]. Antibiotic-induced release of Lipotiechoic acids and peptidoglycan (PG) in case of gram positive bacteria has been shown to enhance systemic inflammatory responses [34]. An endolysin-deficient phage does not degrade the bacterial cell wall, thus progeny are not released until the cell disintegrates or is lysed by other means. However, the phage protein holin, produces an inner membrane lesion at the end of the phage replication cycle, which terminates respiration [7] and ensures killing of the cell. In an *in vivo *situation, we can expect such dead cells to be cleared rapidly by the host immune system.

Non-replicating genetically modified filamentous phage which exerted high killing efficiency on cells with minimal release of endotoxin is reported [13]. Higher survival rate correlated with reduced inflammatory response in case of infected mice treated with genetically modified phage [14]. A phage genetically engineered to produce an enzyme that degrades extracellular polymeric substances and disperses biofilms is reported [15].

Although temperate phages present the problem of lysogeny and the associated risk of transfer of virulence factors through bacterial DNA transduction; we have used a temperate phage as a model for this study as the prophage status simplifies genetic manipulation. Because *S. aureus *strains are known to harbor multiple prophages, which could potentially interfere with recombination and engineering events, we elected to lysogenize phage P954 in a prophage-free host, *S. aureus *RN4220. Our strategy was to identify lysogens that harbored the recombinant endolysin-deficient phages, based on detection of phage P954 genes and the *cat *marker gene by PCR analysis (Figure 1).

In the recombination experiment, the 96 chloramphenicol resistant colonies obtained represented recombinant endolysin-inactivated prophage some of which lysed upon Mitomycin C induction. We suspected that the parent phage could also have lysogenized along with the recombinant phage. We overcame the problem by repeating the induction of chloramphenicol resistant lysogens and lysogenization of the phages produced.

When we assessed the prophage induction pattern and phage progeny release of parent and endolysin-deficient phage P954 lysogens, we found that the absorbance of the culture remained unaltered and the extracellular phage titer was minimal with the recombinant phage lysogen. We observed a low phage titer 3 to 4 hours after induction, presumably due to natural disintegration and lysis of a small percentage of the cell population. In contrast, we observed lysis of the culture by the parent phage with increasing phage titer in the lysate, as expected (Figure 2).

Complementation of the lysis-deficient phenotype was achieved using a heterologous phage P926 from our collection. Supplying the endolysin gene in *trans *allowed the recombinant phage to form plaques (Figure 3b, d). This was used to determine titers of the endolysin-deficient phage throughout our study, and provided an excellent method for efficient phage enrichment. Use of a heterologous phage endolysin enabled the recombinant phage to exhibit the lysis-deficient phenotype even after several rounds of multiplication. *In vitro *activity of the endolysin-deficient phage against MSSA and MRSA was comparable to that of the parent phage (Figure 4). Further, the recombinant phage was able to rescue mice from fatal MRSA infection (Figure 5), similar to the parent phage (data not shown). Future studies will have to compare systemic responses and outcomes of treatment with native and endolysin- deficient phage in *S. aureus *infection.

This work demonstrates the potential of disrupting the endolysin gene to reduce the number of phages that are otherwise released post-infection by their lytic parent phage. In clinical situations, this would provide the advantage of a defined dosage, which is an important concern raised against phage therapy [5,35], as well as lower immune response and reduced endotoxin release when using gram-negative bacteria. This is the first report of a gram-positive endolysin-deficient phage. Our results demonstrate the therapeutic potential of engineered phages in clinical applications.

## Conclusions

We developed a modified bacteriophage against *S. aureus *by insertional inactivation of its endolysin gene, which renders it incapable of host cell lysis. This phage is lethal to cells it infects, with little or no release of progeny phage. We showed that the disrupted endolysin could be complemented with a functional heterologous endolysin gene to produce this phage in high titers. To our knowledge, this is the first report of a gram-positive endolysin-deficient phage. Further, we demonstrate its therapeutic potential in an experimental infection model in mice, in which the lysis-deficient phage P954 protects against lethal MRSA.

## List of Abbreviations

*cat*: Chloramphenicol acetyltransferase; CFU: Colony-forming unit; IP: Intraperitoneal; LB: Luria Bertani; LD_80_: Lethal dose that results in 80% mortality; MOI: Multiplicity of infection; MRSA: Methicillin-resistant *Staphylococcus aureus*; MSSA: Methicillin-sensitive *Staphylococcus aureus*; PFU: Plaque-forming unit

## Competing interests

Authors SP, BS, and JR are inventors on an issued patent (US Patent No 6,896,882) describing the concept of lysin-deficient bacteriophages as therapeutic agents for the control of bacterial infection and methods of developing such phages. Authors have assigned rights to Gangagen Inc., which is a current employer of JR, BS, SSR, SS, and SH and a previous employer of SP, VDP, and NK.

## Authors' contributions

All the authors were affiliated with Gangagen Biotechnologies Pvt. Ltd. when this work was carried out. JR conceived of the concept underlying this study and contributed to the discussion in the manuscript. SP and BS participated in study design and coordination and contributed to data interpretation. VDP, SSR, and SS carried out cloning and generation of the recombinant phage. SH and NK performed *in vivo *studies. VDP and SSR helped draft the manuscript. All authors read and approved the final manuscript.

## Supplementary Material

Additional file 1 **Figure S1 - Genome map of phage P954**. Phage P954 genome is similar in organization to other known temperate staphylococcal phages. The organization of the genome is modular, with genes involved in lysogeny, replication, DNA packaging, tail assembly, and lysis arranged sequentially). Click here for file

Additional file 2 **Table S1 - Comparison of host range of parent and endolysin deficient phage P954**. The host range of both the phage were same on a panel of 20 phage-sensitive and phage-resistant isolates. Click here for file

## References

[B1] Barrow PA Soothill JS Bacteriophage therapy and prophylaxis: rediscovery and renewed assessment of potential Trends Microbiol 1997 5 268 271 10.1016/S0966-842X(97)01054-8 9234508

[B2] Thacker PD Set a microbe to kill a microbe: Drug resistance renews interest in phage therapy JAMA 2003 290 3183 3185 10.1001/jama.290.24.3183 14693857

[B3] Soothill JS Hawkins C Anggard EA Harper DR Therapeutic use of bacteriophages Lancet Infectious Diseases 2004 4 544 545 15336219 10.1016/S1473-3099(04)01127-2

[B4] Lang L FDA approves use of bacteriophages to be added to meat and poultry products Gastroenterology 2006 131 1370 1372 17067600 10.1053/j.gastro.2006.10.012

[B5] William Summers C Bacteriophage therapy Annu Rev Microbiol 2001 55 437 451 10.1146/annurev.micro.55.1.437 11544363

[B6] Young R Bacteriophage lysis: mechanism and regulation Microbiol Rev 1992 56 430 81 1406491 10.1128/mr.56.3.430-481.1992 PMC372879

[B7] Young RJ Bacteriophage holins: deadly diversity Mol Microbiol Biotechnol 2002 4 21 36 11763969

[B8] Loessner MJ Bacteriophage endolysins - current state of research and applications Current Opinion in Microbiology 2005 8 480 487 10.1016/j.mib.2005.06.002 15979390

[B9] Merril CR Biswas B Carlton R Jensen NC Creed GJ Zullo S Adhya S Long-circulating bacteriophage as antibacterial agents Proc Natl Acad Sci 1996 93 3188 3192 10.1073/pnas.93.8.3188 8622911 PMC39580

[B10] Projan S Phage-inspired antibiotics? Nat Biotechnol 2004 22 185 91 10.1038/nbt932 14755287

[B11] Padmanabhan S Sriram B Sagar P Shashikala V Ramachandran J Insertional inactivation of the T4 lysozyme gene: Model for absolute lysis-defectives in phage therapy ASM Conference on the New Phage Biology: the 'Phage Summit':1-5 Aug 2004; Key Biscayne, Florida, USA15720541

[B12] Ramachandran J Sriram P Sriram B Lysin deficient bacteriophages having reduced immunogenecity US Patent No; 6,896,882

[B13] Hagens S Bläsi U Genetically modified filamentous phage as bactericidal agents: a pilot study Lett Appl Microbiol 2003 37 318 323 10.1046/j.1472-765X.2003.01400.x 12969496

[B14] Hagens S Habel A von Ahsen U von Gabain A Bläsi U Therapy of experimental pseudomonas infections with a nonreplicating genetically modified phage Antimicrob Agents Chemother 2004 48 3817 3822 10.1128/AAC.48.10.3817-3822.2004 15388440 PMC521880

[B15] Lu TK Collins JJ Dispersing biofilms with engineered enzymatic bacteriophage Proc Natl Acad Sci 2007 104 11197 11202 10.1073/pnas.0704624104 17592147 PMC1899193

[B16] Matsuda T Freeman TA Hilbert DW Duff M Fuortes M Stapleton PP Daly JM Lysis-deficient bacteriophage therapy decreases endotoxin and inflammatory mediator release and improves survival in a murine peritonitis model Surgery 2005 137 639 646 10.1016/j.surg.2005.02.012 15933632

[B17] Hiramatsu K Katayama Y Yuzawa H Ito T Molecular genetics of methicillin-resistant Staphylococcus aureus Int J Med Microbiol 2002 292 67 74 10.1078/1438-4221-00192 12195737

[B18] Smith TL Pearson ML Wilcox KR Cruz C Lancaster MV Robinson-Dunn B Tenover FC Zervos MJ Band JD White E Jarvis WR Emergence of vancomycin resistance in Staphylococcus aureus. Glycopeptide-Intermediate Staphylococcus aureus Working Group N Engl J Med 1999 340 493 501 10.1056/NEJM199902183400701 10021469

[B19] CDC Staphylococcus aureus Resistant to Vancomycin - United States 2002 MMWR 2002 51 565 567 12139181

[B20] Perl TM Golub JE New approaches to reduce Staphylococcus aureus nosocomial infection rates: treating *S. aureus *nasal carriage Ann Pharmacother 1998 32 S7 16 9475834 10.1177/106002809803200104

[B21] Klevens RM Morrison MA Nadle J Petit S Gershman K Ray S Harrison LH Lynfield R Dumyati G Townes JM Craig AS Zell ER Fosheim GE McDougal LK Carey RB Fridkin SK Invasive methicillin-resistant Staphylococcus aureus infections in the United States JAMA 2007 298 1763 1771 10.1001/jama.298.15.1763 17940231

[B22] Merril CR Scholl D Adhya SL The prospect for bacteriophage therapy in Western medicine Nat Rev Drug Discov 2003 2 489 497 10.1038/nrd1111 12776223

[B23] Kreiswirth BN Löfdahl S Betley MJ O'Reilly M Schlievert PM Bergdoll MS Novick RP The toxic shock syndrome exotoxin structural gene is not detectably transmitted by a prophage Nature 1983 305 709 712 10.1038/305709a0 6226876

[B24] Mahmood R Khan SA Role of upstream sequences in the expression of the Staphylococcal enterotoxin B gene J Biol Chem 1990 265 4652 4656 2307681

[B25] Lee CY Cloning of genes affecting capsule expression in Staphylococcus aureus strain M Mol Microbiol 1992 6 1515 1522 10.1111/j.1365-2958.1992.tb00872.x 1625580

[B26] Jankovic I Egeter O Brückner R Analysis of catabolite control protein A-dependent repression in Staphylococcus xylosus by a genomic reporter gene system J Bacteriol 2001 183 580 586 10.1128/JB.183.2.580-586.2001 11133951 PMC94913

[B27] Adams MH Bacteriophages 1959 New York: Interscience Publishers

[B28] Carlson K Kutter E, Sulakvelidze A Working With Bacteriophages: Common Techniques And Methodological Approaches Bacteriophages: Biology and Applications 2005 CRC press 437 490

[B29] Sambrook J Russel DW Molecular Cloning: A Laboratory Manual 2001 3 Cold Spring Harbor Laboratory Press. Cold Spring Harbor, New York

[B30] Schenk S Laddaga RA Improved method for electroporation of Staphylococcus aureus FEMS Microbiol Lett 1992 73 133 138 1521761 10.1016/0378-1097(92)90596-g

[B31] Altschul SF Gish W Miller W Myers EW Lipman DJ Basic local alignment search tool J Mol Biol 1990 215 403 410 2231712 10.1016/S0022-2836(05)80360-2

[B32] Lukashin A Borodovsky M GeneMark.hmm: new solutions for gene finding Nucleic Acids Research 1998 26 1107 1115 10.1093/nar/26.4.1107 9461475 PMC147337

[B33] Shenep JL Barton RP Mogan KA Role of antibiotic class in the rate of *liberation of endotox*in during therapy for experimantal gram-negative bacterial sepsis J Infect Dis 1985 151 1012 1018 10.1093/infdis/151.6.1012 3889171

[B34] Van Langevelde P Ravensbergen E Grashoff P Beekhuizen H Groeneveld PH Van Dissel JT Antibiotic-induced cell wall fragments of Staphylococcus aureus increase endothelial chemokine secretion and adhesiveness for granulocytes Antimicrob Agents Chemother 1999 43 2984 2989 10582893 10.1128/aac.43.12.2984 PMC89598

[B35] Schoolnik GK Summers WC Watson JD Phage offer a real alternative Nature Biotechnol 2004 22 505 506 10.1038/nbt0504-505 15122279

