# Supplementary material for: Lysis-deficient phages as novel therapeutic agents for controlling bacterial infection
Source: BMC Microbiol. 2011 Aug 31;11:195. doi: 10.1186/1471-2180-11-195 (PMC3224134; doi:10.1186/1471-2180-11-195)

**Additional file 1, Figure S1 – Genome map of phage P954**

Phage P954 genome is similar in organization to other known temperate staphylococcal phages. The organization of the genome is modular, with genes involved in lysogeny, replication, DNA packaging, tail assembly, and lysis arranged sequentially).


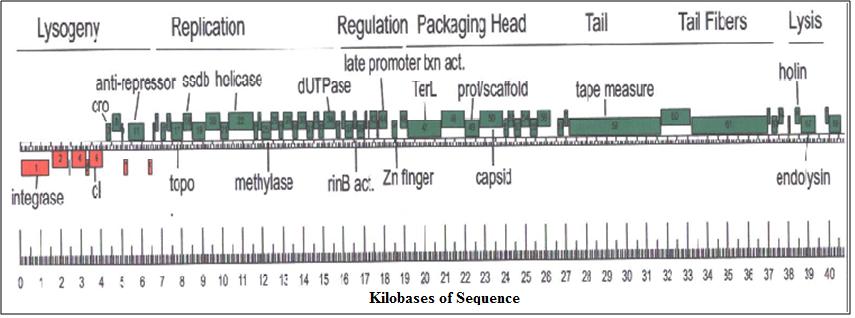

Supplement: Additional file 1 — Figure S1 - Genome map of phage P954. Phage P954 genome is similar in organization to other known temperate staphylococcal phages. The organization of the genome is modular, with genes involved in lysogeny, replication, DNA packaging, tail assembly, and lysis arranged sequentially). [file 1471-2180-11-195-S1.DOC]
