# Supplementary material for: Lysis-deficient phages as novel therapeutic agents for controlling bacterial infection
Source: BMC Microbiol. 2011 Aug 31;11:195. doi: 10.1186/1471-2180-11-195 (PMC3224134; doi:10.1186/1471-2180-11-195)
Supplement: Additional file 2 — Table S1 - Comparison of host range of parent and endolysin deficient phage P954. The host range of both the phage were same on a panel of 20 phage-sensitive and phage-resistant isolates. [file 1471-2180-11-195-S2.DOCX]

**Additional file 1, Table S1 - Comparison of host range of parent and endolysin deficient phage P954**

The host range of both the phage were same on a panel of 20 phage-sensitive and phage-resistant isolates

| **Strain** | **Methicillin sensitivity** | **Endolysin deficientP954** | **Wild type P954** |
| --- | --- | --- | --- |
| B911 | MSSA | + | + |
| B9007 | MSSA | + | + |
| B9030 | MSSA | − | − |
| B9160 | MSSA | − | − |
| B9161 | MSSA | − | − |
| B9165 | MSSA | − | − |
| B9166 | MSSA | − | − |
| B9167 | MSSA | − | − |
| B9189 | MSSA | + | + |
| B9204 | MSSA | − | − |
| RN4220 | MSSA | + | + |
| B910 | MRSA | + | + |
| B954 | MRSA | + | + |
| B9053 | MRSA | + | + |
| B9159 | MRSA | − | − |
| B9162 | MRSA | + | + |
| B9164 | MRSA | − | − |
| B9186 | MRSA | + | + |
| B9194 | MRSA | + | + |
| B9195 | MRSA | + | + |

(+) indicates sensitive

(−) indicates insensitive
